# *N*-acetylcysteine does not prevent contrast-induced nephropathy after cardiac catheterization in patients with diabetes mellitus and chronic kidney disease: a randomized clinical trial

**DOI:** 10.1186/1745-6215-10-45

**Published:** 2009-06-29

**Authors:** Manouchehr Amini, Mojtaba Salarifar, Alireza Amirbaigloo, Farzad Masoudkabir, Fatemeh Esfahani

**Affiliations:** 1 Department of nephrology, Shariati Hospital, Tehran University of Medical Sciences, Tehran, Iran; 2 Department of cardiology, Tehran Heart Center, Tehran University of Medical Sciences, Tehran, Iran; 3 Research Development Center, Shariati Hospital, Tehran University of Medical Sciences, Tehran, Iran; 4 Student Scientific Research Center (SSRC), Tehran University of Medical Sciences, Tehran, Iran

## Abstract

**Background:**

Patients with diabetes mellitus (DM) and chronic kidney disease (CKD) constitute to be a high-risk population for the development of contrast-induced nephropathy (CIN), in which the incidence of CIN is estimated to be as high as 50%. We performed this trial to assess the efficacy of *N*-acetylcysteine (NAC) in the prevention of this complication.

**Methods:**

In a prospective, double-blind, placebo controlled, randomized clinical trial, we studied 90 patients undergoing elective diagnostic coronary angiography with DM and CKD (serum creatinine ≥ 1.5 mg/dL for men and ≥ 1.4 mg/dL for women). The patients were randomly assigned to receive either oral NAC (600 mg BID, starting 24 h before the procedure) or placebo, in adjunct to hydration. Serum creatinine was measured prior to and 48 h after coronary angiography. The primary end-point was the occurrence of CIN, defined as an increase in serum creatinine ≥ 0.5 mg/dL (44.2 μmol/L) or ≥ 25% above baseline at 48 h after exposure to contrast medium.

**Results:**

Complete data on the outcomes were available on 87 patients, 45 of whom had received NAC. There were no significant differences between the NAC and placebo groups in baseline characteristics, amount of hydration, or type and volume of contrast used, except in gender (male/female, 20/25 and 34/11, respectively; P = 0.005) and the use of statins (62.2% and 37.8%, respectively; P = 0.034). CIN occurred in 5 out of 45 (11.1%) patients in the NAC group and 6 out of 42 (14.3%) patients in the placebo group (P = 0.656).

**Conclusion:**

There was no detectable benefit for the prophylactic administration of oral NAC over an aggressive hydration protocol in patients with DM and CKD.

**Trial registration:**

NCT00808795

## Background

Contrast-induced nephropathy (CIN) is the third most common cause of hospital acquired acute kidney injury, accounting for 10% of all cases[1]. With the increasing use of contrast media in diagnostic and interventional procedures, it has become one of the major challenges encountered during routine cardiovascular practice. Generally, this form of acute kidney injury follows a benign course and only rarely necessitates use of dialysis [2-4]. Nevertheless, use of radiocontrast media has been associated with increased in-hospital morbidity, mortality, and costs of medical care, long admission, especially in patients needing dialysis [5-8]. Patients at the greatest risk for CIN can be defined as those that have preexisting impaired renal function and diabetes mellitus with the incidence estimated to be as high as 50% [9]. Therefore, these patients constitute to be an appropriate target population for efforts at prevention of this important complication. Preventive therapies primarily include limitation of contrast exposure, intravenous volume expansion with a saline solution, and use of low- or iso-osmolality contrast media [10]. However, since these measures provide incomplete protection for CIN, interest has emerged in a number of adjunction short-term pharmacotherapy methods. Among them, *N*-acetylcysteine (NAC) has been of considerable interest after an initial report by Tepel et al. [11]. They showed a reduction in the incidence of CIN with NAC compared with hydration alone. Up to now, several clinical studies [9,12-26] and meta-analyses [27-37] have been performed to assess the efficacy of NAC in the prevention of CIN, but the results are widely controversial even among the meta-analyses. In spite of heterogeneity in the available data on the efficacy of NAC, several studies have advised the use of NAC, especially in high-risk patients, because of its low cost, availability, and few side effects. It, however, seems that we need more evidence about the efficacy and cost-effectiveness of NAC in patients at high risk for the development of CIN to make rational clinical decisions for individual patients as well as policy decisions for the health of the general public.

The purpose of this study was to extend our understanding of the potentials of NAC in the prevention of CIN in patients with diabetes mellitus and chronic kidney disease.

## Methods

### Study patients

Between April 2006 and October 2006, ninety consecutive eligible patients scheduled for elective diagnostic coronary angiography at the cardiac catheterization laboratory of "Tehran Heart Center" (Tehran University of Medical Sciences) were enrolled in this study. We included patients older than 18 years old with a history of diabetes mellitus for at least one year and chronic kidney disease, defined as serum creatinine concentration ≥ 1.5 mg/dL for men and ≥ 1.4 mg/dL for women. Patients with acute coronary syndrome requiring primary or rescue coronary intervention within less than 12 h, cardiogenic shock, current peritoneal or hemodialysis, or a known allergy to NAC were excluded from the study. The study protocol was approved by the ethics committees of Tehran University of Medical Sciences and Tehran Heart Center, and written informed consent was obtained from all the patients.

### Study protocol

The study was a prospective, double-blind, placebo controlled, randomized clinical trial. The patients were randomly assigned on a 1:1 fashion via the balanced block randomization method using computer generated random numbers to receive NAC or placebo by randomly drawing sealed envelopes containing either the active drug or matching placebo. NAC and placebo were prepared by Darmanyab Co. (agency of Zambon Group S.p.A, Milan, Italy) matched in appearance, packing, and way of use. NAC was orally administered at the dose of 600 mg twice a day, starting 24 h before the procedure (two doses before and two doses after the procedure). The patients were hydrated orally and intravenously. All the patients were encouraged to drink fluids like water and fruit juice for at least 8 glasses over 12 h before the procedure and memorize the number of glasses. The oral pre-procedural hydration was estimated by multiplying the number of glasses drunk by 200 mL (estimated volume of a glass). In addition, the patients were hydrated intravenously by 1 L of 0.9 normal saline, which was commenced in the catheterization laboratory. Serum creatinine and urea nitrogen concentrations were measured prior to coronary angiography and 48 h after the procedure. Serum creatinine concentration prior to coronary angiography was referred to as the baseline level. Creatinine clearance (CrCl) was estimated with the Cockcroft-Gault formula, where CrCl = ([140-age]*weight(kg)/serum creatinine(mg/dL)*72), with adjustment for female sex (CrCl_female _= CrCl*0.85)[38]. Coronary angiographies were performed with the low osmolar nonionic contrast medium Iohexol (Omnipaque; Amersham Health, Co. Cork, Ireland) or the iso-osmolar nonionic contrast medium Iodixanol (Visipaque;GE Healthcare, Co. Cork, Ireland) and/or the high osmolar ionic medium Diatrizoate meglumine/sodium (Urografin; Schering AG, Berlin, Germany).

### End-points

The primary end-point of the study was the occurrence of CIN, defined as an increase in serum creatinine concentration ≥ 0.5 mg/dL(44.2 μmol/L) or ≥ 25% above the baseline at 48 h after exposure to the contrast medium[5,11]. The secondary end-points were: (1) the change in serum creatinine 48 h after exposure to the contrast agent; (2) the change in serum urea nitrogen 48 h after the procedure; and (3) the change in CrCl 48 h after coronary angiography.

### Statistical analysis

According on the study of Tepel et al[11], a sample size of 42 patients in each group would be sufficient to detect a difference of 19% between the groups in the rate of CIN at 48 h after exposure to the contrast medium, with 80% power and a 5% significance level. This 19% difference represents the difference between a 21% CIN rate in the placebo group and a 2% rate in the treatment group. This number has been increased to 45 per group to allow for a predicted drop-out from treatment of around 5%.

Data distribution was checked by histogram and the Kolmogorov-Smirnov test.

The continuous data were expressed as mean ± SD and were compared via the Student t-test. The categorical data were expressed as number and percentage and were compared via the Chi-square test or Fischer exact test. Two tailed P < 0.05 was considered significant. The data were analyzed with SPSS software, version 13.0 (SPSS Inc, Chicago, Illinois, USA).

## Results

### Patients

Of the 90 patients enrolled in the study, 3 patients in placebo group were lost to follow-up because of immediate hospital discharge after coronary angiography and failure to have subsequent blood sampling performed. Thus, only 42 patients were evaluable for the assessment of the outcomes in the placebo group. We present the baseline clinical, pharmacological, and laboratory characteristics of the study patients in Table 1.

**Table 1:** Baseline clinical, pharmacological, and laboratory characteristics of study patients^a^

	NAC group(n = 45)	Placebo group(n = 45)	*P *value
Baseline clinical characteristics			
Age, years	63.25 ± 9.78	65.09 ± 9.40	0.45
Sex (male/female)	20/25	34/11	0.005
Body mass index, kg/m^2^	28.56 ± 4.81	27.85 ± 4.13	0.45
Time since diagnosis of DM, years	10.11 ± 8.50	9.82 ± 7.57	0.86
Systolic blood pressure, mmHg	156.05 ± 19.9	151.95 ± 18.4	0.34
Diastolic blood pressure, mmHg	82.63 ± 7.60	81.22 ± 7.14	0.39
Ejection fraction, %	49.45 ± 10.99	46.83 ± 14.15	0.37
Medications			
Aspirin, n(%)	37(82.2%)	36(80%)	1.00
ACE-inhibitors/ARB, n(%)	32(71.1%)	30(66.7%)	0.82
β-blockers, n(%)	29(64.4%)	35(77.8%)	0.24
Nitrates, n(%)	30(66.7%)	30(66.7%)	1.00
Diuretics, n(%)	23(51.1%)	19(42.2%)	0.52
Statins, n(%)	28(62.2%)	17(37.8%)	0.034
Digitals, n(%)	6(13.3%)	6(13.3%)	1.00
Baseline laboratory values			
Serum creatinine, mg/dL	1.736 ± 0.42	1.736 ± 0.17	1.00
Serum urea nitrogen, mg/dL	60.56 ± 28.37	58.64 ± 28.94	0.75
Creatinine clearance, mL/min	42.76 ± 11.97	43.97 ± 11.91	0.63

^a ^All plus-minus values are mean ± SD. DM, diabetes mellitus; ACE, angiotensin converting enzyme; ARB, angiotensin receptor blockers.

There were no significant differences between the treatment groups with regard to CHD risk factors, baseline serum creatinine, and urea nitrogen concentration or CrCl except for the gender, which was significantly different between the two groups of patients (*P *= 0.005). Also, with respect to concomitant medications, there were no significant differences between the NAC group and placebo group except in the use of statins (62.2% versus 37.8% respectively, *P *= 0.034). Cardiac catheterization data, consisting of type and dose of the contrast agents, are shown in Table 2. Since 22 patients received a combination of Diatrizoate meglumine/sodium with Iohexol or Iodixanol, we also calculated the total dose of contrast in each group. There were no significant differences between the two groups in regard to the type and dose of the radiocontrast agents administered for coronary angiography (*P *for all > 0.05).

**Table 2:** Cardiac catheterization data^a^

	NAC group (n = 45)	Placebo group (n = 45)	*P *value
Preprocedural hydration, mL	2254 ± 633	2272 ± 602	0.89
Type of radiocontrast agent			
Iohexol, n(%)	32(72%)	34(76%)	1.00
Iodixanol, n(%)	1(2%)	0(0%)	1.00
Diatrizoate meglumine/sodium, n(%)	0(0%)	1(2%)	0.88
Diatrizoate meglumine/sodium+Iodixanol, n(%)	1(2%)	1(2%)	1.00
Diatrizoate meglumine/sodium+Iohexol, n(%)	11(24%)	9(20%)	0.82
Dose of radiocontrast agent			
Iohexol, mL	100.11 ± 37.80	102.22 ± 40.43	0.37
Iodixanol, mL	6.11 ± 28.78	1.56 ± 10.43	0.32
Diatrizoate meglumine/sodium, mL	11.78 ± 20.48	17.33 ± 36.51	0.79
Total, mL	118.00 ± 35.20	121.11 ± 43.95	0.71

^a ^All plus-minus values are mean ± SD.

### Primary end-point

CIN, defined as an increase in serum creatinine concentration of ≥ 0.5 mg/dL or ≥ 25% above the baseline, was not significantly different between the NAC and placebo groups (5/45 [11.1%] vs. 6/42 [14.3%], respectively; relative risk: 0.78 [95% CI: 0.26–2.36]; *P *= 0.656).

### Secondary end-point

No difference was observed between the groups regarding the secondary end-points. The changes in serum creatinine, serum urea nitrogen, and CrCl 48 h after coronary angiography were similar between the groups. The data are presented in Table 3.

**Table 3:** Primary and secondary end-points after coronary angiography^a^

	NAC group(n = 45)	Placebo group(n = 45^b^)	*P *value
Primary end-point			
Incidence of CIN, n(%)	5(11.1%)	6(14.3%)	0.656
Secondary end-points			
Change in serum creatinine, mg/dL	-0.016 ± 0.363	-0.018 ± 0.467	0.975
Change in serum urea nitrogen, mg/dL	-1.73 ± 24.03	-3.71 ± 24.26	0.703
Change in creatinine clearance, mL/min	2.86 ± 8.84	3.78 ± 10.87	0.676

^a ^All plus-minus values are mean ± SD. CIN, contrast-induced nephropathy.

^b^While 45 patients were enrolled in each group, serum creatinine and urea nitrogen measurements were not available for 3 placebo patients who were discharged immediately after coronary angiography and did not return for subsequent blood sampling (comparison, 45 NAC patients vs. 42 placebo patients).

## Discussion

The potential of NAC to reduce the risk of CIN has been a topic of intense and recent interest, manifested by the number of prospective clinical trials on this topic [9,12-26]. This is likely, in part, due to the absence of effective adjunctive pharmacotherapy methods for this important complication. However, it seems likely that the potential for benefit from NAC, low cost, and the absence of considerable data indicating potential harm also have contributed to make NAC advisable, not least in high-risk patients, before a definitive demonstration of meaningful clinical benefit on the incidence of CIN and its morbidity and mortality.

In this study, we evaluated the efficacy of NAC exclusively in high-risk patients for the development of CIN. The main finding of the current study was that the prophylactic oral administration of NAC did not provide any benefit as compared with a placebo to reduce the incidence of CIN in patients with chronic kidney disease and diabetes mellitus, who are a high-risk population for the development of CIN. Our findings are consistent with those in studies reporting that NAC provides no benefit over hydration for the prevention of CIN [12,13,15,17,20,22,23]. In addition, our study supports and expands the studies by Coyle et al[26], Durham et al. [17], and Gomes et al. [9,24], who evaluated the efficacy of NAC in the prevention of CIN in diabetic patients. They concluded that NAC provided no benefit over an aggressive hydration protocol in this population and also suggested that this intervention could even be harmful. But, on the other hand, there are several clinical studies reporting findings that do not chime with ours [9,16,18,19,21,22,25]. Previously, a post-hoc analysis of a subgroup of 75 diabetic patients [21] indicated that NAC might effectively prevent CIN in patients with diabetes mellitus but our study did not confirm this finding.

What are we to make of such conflicting results? Fishbane et al. [39]compared positive and negative studies and noted that the studies showing no benefit for NAC had a much lower incidence of CIN in the placebo group than did those studies showing NAC to be beneficial (11.0% compared to 24.8%). These results suggest that perhaps NAC is only beneficial for those at a high risk of CIN. Be that as it may, in this study we were unable to show a benefit resulting from the prophylactic administration of oral NAC in a high-risk group of patients with diabetes mellitus and chronic renal insufficiency (mean baseline creatinine 1.74 mg/dL).

The incidence of CIN is currently estimated to be as high as 40–50% amongst patients with diabetes mellitus and preexisting renal disease [6,9,16]. In this study, the overall incidence of CIN was 12.6%, which is significantly lower than that in previous reports. The low incidence of CIN in our study may have several reasons: (1) our patients were hydrated orally by a mean volume of 2267 ± 645 mL of fluids over the 12-h period before the procedure followed by 1 L of IV 0.9 normal saline beginning in the catheterization laboratory. In comparison, the other studies reporting a higher incidence of CIN, have typically used lower amounts of hydration [9,40,41], which may be insufficient for maximal protection from contrast nephrotoxicity. (2) The mean dose of contrast agents used in our study was lower than that of other studies. Over 95% of the patients in our study received Iohexol at least in part and the mean total dose of Iohexol used in our study was about 100 mL, while it has typically been used by amounts of 140 mL to 280 mL in the previous studies [15,25,42].

### Limitations

Several limitations should be noted. The current study protocol excluded patients with acute coronary syndrome, requiring primary or rescue coronary intervention within the first 12 h and cardiogenic shock, and therefore the effect of NAC was not explored in these patient subsets. The relatively small sample size of this study calls for caution interpreting the results. This sample size was predetermined from a power calculation based on the findings of Tepel et al[11]. They found a 19% difference in the rate of CIN between NAC and placebo groups, which was more extreme than what others have cited in favor of NAC. Another potential limitation of this study is that, although there were no significant differences between the NAC group and placebo group with regard to the type of the contrast agents used, the multiplicity of the type of the contrast agents was a potential limitation of this study.

## Conclusion

Our major finding was that NAC had no advantage over an aggressive hydration protocol in patients with diabetes mellitus and chronic renal insufficiency undergoing diagnostic coronary angiography. On the basis of these findings, we believe that the use of NAC to prevent CIN in this population should not be encouraged. Our findings support that the recommended measures for preventing CIN continue to be appropriate hydration, even greater than the standard regimen for hydration, and the use of a small volume of contrast in patients with a high risk of CIN undergoing coronary angiography.

## Abbreviations

CIN: contrast-induced nephropathy; CKD: chronic kidney disease; DM: diabetes mellitus; *NAC*: *N*-acetylcysteine.

## Competing interests

The authors declare that they have no competing interests.

## Authors' contributions

MA was responsible for trial designing, supervision of the trial implementation, and writing the article. MS was responsible for trial designing and angiographic studies. AA was responsible for study design, trial implementation, visiting patients, patients' follow-up, and writing the article. FM was responsible for epidemiologic consultation, statistical analysis, and writing the article. FE was responsible for epidemiologic consultation and statistical analysis.
